# Estimating the best PCSK9 vaccine design for atherosclerosis based on mouse models: A frequentist network meta-analysis

**DOI:** 10.21542/gcsp.2025.19

**Published:** 2025-05-15

**Authors:** Muhammad Iqhrammullah, Asyraf Muzaffar, Derren David Christian Homenta Rampengan, Chairul Ichwan, Ayers Gilberth Ivano Kalaij, Seba Talat Al-Gunaid, Nur Adiba Purba, Naufal Gusti, Muhammad Rinaldi Sufri, Adi Purnawarman

**Affiliations:** 1 Postgraduate School of Public Health, Universitas Muhammadiyah Aceh, Banda Aceh, Indonesia; 2 School of Medicine, Universitas Syiah Kuala, Banda Aceh, Indonesia; 3 Faculty of Medicine, Universitas Sam Ratulangi, Manado, Indonesia; 4 Department of Cardiology and Vascular Medicine, Dr. Zainoel Abidin Hospital, Banda Aceh, Indonesia; 5 Faculty of Medicine, Universitas Indonesia, Jakarta, Indonesia; 6 Department of Cardiology and Vascular Medicine, Dr. Soetomo Hospital, Faculty of Medicine, University of Airlangga, Surabaya, Indonesia; 7 Directoral General of Disease Control and Prevention, Ministry of Health, Jakarta, Indonesia; 8 Department of Cardiology and Vascular Medicine, Faculty of Medicine, Universitas Syiah Kuala, Banda Aceh, Indonesia

## Abstract

**Background:** Proprotein convertase subtilisin/kexin 9 (PCSK9) plays a crucial role in regulating the plasma levels of low-density lipoprotein cholesterol (LDL-C) and low-density lipoprotein receptor (LDLR). In contrast, vaccines have been developed to induce PCSK9-specific antibodies, which could improve dyslipidemia in atherosclerotic animal models. The aim of this study was to determine the best PCSK9 vaccine design for improving dyslipidemia and atherosclerosis in animal studies.

**Methods:** A systematic search was performed on PubMed, Scopus, and Web of Science for studies published as of August 2024. Studies that developed a PCSK9 vaccine and reported its efficacy in mouse models were included. A random-effects model was used to conduct a frequentist network meta-analysis. The standardized mean difference (SMD) and 95% confidence interval (CI) were used as size effects. Heterogeneity in the model was judged based on the *I*^2^ values.

**Results:** Six studies, published between 2014 and 2024, were included. There were 8 vaccine designs developed using different peptides, carriers, and delivery systems. Vaccines prepared with human PCSK9-003 epitope peptide (F-A-Q-S-I-P-W-N) with Qb virus-like particles consistently appeared to be the best vaccine design for the reduction of total cholesterol (*p* < 0.001), LDL-C (*p* = 0.002), and triglycerides (*p* = 0.024). The heterogeneity was low for total cholesterol and LDL-C (*I^2^* = 16% and 0%, respectively) and moderate for triglycerides (*I^2^* =64%).

**Conclusion:** Vaccines prepared from the PCSK9-003 epitope peptide (F-A-Q-S-I-P-W-N) conjugated with Qb virus-like particles are suggested to be promising for atherosclerosis treatment by reducing LDL-C and improving other lipid profiles.

**PROSPERO:** CRD42024596892.

## Introduction

Atherosclerosis remains the primary cause of morbidity and mortality worldwide and serves as a common etiology of cardiovascular disease^1–3^. Chronic inflammation plays a crucial role in the development and progression of atherosclerosis, ultimately resulting in plaque rupture and erosion, leading to atherosclerosis-related cardiovascular diseases^4^. Various genetic and acquired atherosclerosis risk factors can damage and modify the normal homoeostatic functions of the endothelium, triggering an inflammatory response^5^. According to data from the American College of Cardiology, the age-specific mortality rate for cardiovascular disease ranges from 73.6 per 100,000 in the high-income Asia Pacific to 432.3 per 100,000 in Eastern Europe in 2022^6^. Atherosclerosis-related events such as intracerebral hemorrhage and ischemic stroke remain the leading causes of cardiovascular disease mortality worldwide^2^.

Among the primitive forms, hypercholesterolemia is the most common cause of early cardiovascular disease (CVD) owing to continuous exposure to dyslipidemia^5^. Currently, statin therapy is the gold standard treatment for hypercholesterolemia and has been shown to improve cardiac outcomes^7–9^. Statins are effective in inhibiting 3-hydroxy-3-methyl-glutaryl-coenzyme A (HMG-CoA) reductase, which decreases the production of low-density lipoprotein cholesterol (LDL-C) and has been shown to significantly reduce complications^10^. However, statin therapy is still insufficient to achieve LDL-C targets in some high-risk individuals, such as those with a history of cardiovascular events, familial hypercholesterolemia, or persistently elevated LDL-C levels despite maximum tolerated statin doses^11^. Moreover, in individuals with secondary dyslipidemia, statins may be insufficient to achieve the target LDL-C levels due to disrupted lipid metabolism. Additionally, some individuals may not respond to statins owing to genetic factors. In cases of elevated apolipoprotein(a), statin therapy generally has little to no impact. A meta-analysis of multiple countries reported that the prevalence of statin intolerance could reach 9.1%^12^. Therefore, more potent LDL-lowering medications are typically required for individuals who require substantial LDL-C reduction.

LDL-C may act as an independent contributor to atherosclerosis^13,14^. The main pathway for clearance of LDL-C from the bloodstream is its uptake by the liver LDL receptor (LDLR)^14,15^. Proprotein convertase subtilisin/kexin 9 (PCSK9) is a serine protease that binds LDLR and functions in lysosomal degradation^16–18^. This action effectively blocks the recirculation of LDLR to the cell surface, thereby increasing circulating LDL-C levels^19^. Inhibition of PCSK9 with monoclonal antibodies (mAbs) has become a cornerstone of lipid-lowering therapy in recent decades and has received particular attention in preventing and managing atherosclerosis and later cardiovascular disease^20,21^. A systematic review and meta-analysis suggested that this approach could significantly improve overall lipid profiles, particularly LDL-C levels^22^. Furthermore, the FOURIER Trial revealed that a proprotein convertase subtilisin/kexin 9 (PCSK9) inhibitor may reduce apolipoprotein(a) levels by 26.9%^23^. Unfortunately, poor adherence to medication is the greatest challenge in lipid-lowering therapies. To overcome this, researchers have pursued a relatively new approach to treating hyperlipidemia through vaccination^24^.

Several studies have been reported, such as one experimental study showed that the L-IFPTA vaccine induces safe, long-lasting, and functional PCSK9-specific antibodies in mice with hypercholesterolemia, and this vaccine produces long-term therapeutic effects as reported in other studies^25^. In another study, the PCSK9 vaccine was reported to significantly reduce plaque lesion area in the aorta and macrophage infiltration in a mouse model of atherosclerosis^26^. Active immunization against PCSK9 has been shown to produce positive effects in mouse models using the AT04A vaccine, which mimics the N-terminal epitope of the human PCSK9 protein and its mouse homologue^27^. This resulted in a significant reduction in serum PCSK9 levels, LDL cholesterol, necrotic core content, and both the area and number of atherosclerotic lesions^27^. With the development of many PCSK9 vaccines, it is imperative to perform a network meta-analysis to comparatively analyze which vaccine has the highest potential.

## Methods

### Study design

This systematic review and network meta-analysis (NMA) was performed using a frequentist approach to compare the effectiveness of various PCSK9-targeted vaccines in improving lipid profiles, specifically total cholesterol, LDL cholesterol (LDL-C), and triglyceride levels, based on the Preferred Reporting Items for Systematic Reviews and Network Meta-Analyses (PRISNMA) guidelines. The protocol was registered in PROSPERO (no. CRD42024596892) and officially accepted as of November 20, 2024. Ethical approval was not required for this study as it was a secondary analysis of published data. All data were obtained from peer-reviewed studies that had received ethical approval.

### Search strategy

A comprehensive literature search was conducted using the Scopus, PubMed, and Web of Science databases from inception to August 2024. The keywords and Medical Subject Headings (MeSH) terms utilized in the search included those related to PCSK9 vaccines and lipid profiles, such as “PCSK9 vaccine,” “lipid profile,” “cholesterol,” “LDL-C,” “triglycerides,” and “network meta-analysis.” The complete keyword combi-nation is as follow: (“Proprotein convertase subtilisin/kexin type 9” OR “dyslipidemia” OR “lipid-lowering” “hypercholesterolemia” OR “atherosclerosis” OR “cholester-ol-lowering” OR PCSK9) AND (vaccine OR immunization). No restrictions were imposed on the language or date.

### Data eligibility criteria

Relevant studies were conducted in accordance with the Population, Intervention, Comparator, Outcome, and Study design (PICOS) framework. The population were experimental studies conducted on murine or simian subjects exhibiting dyslipidemia or an elevated risk of developing cardiovascular disease, the intervention were PCSK9-targeted vaccines, which include PCSK9Qb-003, L-IFPTA+, IFPT, IFPTA+, L-IFPT, V1_5ug, V2_5ug, V1_50ug, and anti-PCSK9, the comparator groups comprised control subjects or placebo recipients, and the principal outcomes of interest were alterations in the lipid profile, specifically in terms of total cholesterol, LDL-C, and triglycerides. Studies that did not clearly indicate the use of mice with dyslipidemia or an increased risk of cardiovascular disease were excluded from this review. Furthermore, studies lacking extractable data and those published as reviews, case reports, editorials, or conference papers were excluded.

### Data collection

Initial searches and screening were carried out according to the PRISMA statement. Duplicates were then removed using EndNotes v9.0 (Clarivate Analytics, Philadelphia, PA, USA). Three independent reviewers (MI, AM, and AGIK) undertook a dual-stage screening process based on the PRISMA Statement, initially on titles and abstracts, and subsequently on full texts of the retrieved articles. This was conducted in accordance with the predetermined eligibility criteria. Discrepancies were then identified by all reviewers until a consensus was reached.

### Quality appraisal

The risk of bias in this review was assessed using a modified CAMARADES (Collaborative Approach to Meta-Analysis and Review of Animal Data from Experimental Studies) checklist. This was designed to ensure the methodological quality of the included studies. The checklist is based on 10 specific criteria: (1) publication in a peer-reviewed journal, (2) statement of temperature control, (3) random allocation to treatment or control, (4) blinding of caregivers or investigators, (5) blinding of outcome assessments, (6) use of co-interventions or presence of co-morbidities, (7) appropriate animal model (in terms of age, sex, species and strain), (8) calculation of sample size, (9) compliance with animal welfare regulations, and (10) statement of potential conflicts of interest.

### Data extraction

Data from the included studies were extracted in the form of study characteristics and study outcome tables. Data extracted from the eligible studies included (1) study characteristics such as author, year, population, sample size, and study design; (2) details of the intervention, including the type of PCSK9 vaccine and dosage regimen; and (3) outcomes, such as mean changes in total cholesterol, LDL-C, and triglycerides, as well as standard deviations or confidence intervals. The data were extracted by M.I. and double-checked by A.M. and A.G.I.K.

### Statistical analysis

Data extracted from the included studies were further analyzed through frequentist network meta-analysis, performed on RStudio version 4.3.2, using the netmeta package. A random-effects model was used to account for potential heterogeneity between trials. Size effects used in the network pooled estimate were standardized mean differences (SMDs) with 95% confidence intervals (CIs) for each comparison. Heterogeneity was quantified using *I*^2^ statistics, where values of 25%, 50%, and 75% were indicative of low, moderate, and high heterogeneity, respectively. Q-statistics were also used to determine heterogeneity (within designs) and inconsistency (between designs). The control group was used as the primary reference in the initial analysis, where the best vaccine was used as the reference in the next analysis. We extended our investigation by carrying out the ranking analysis using the netrank function in the same package (netmeta).

## Results

### Search and selection results

**Figure 1.**
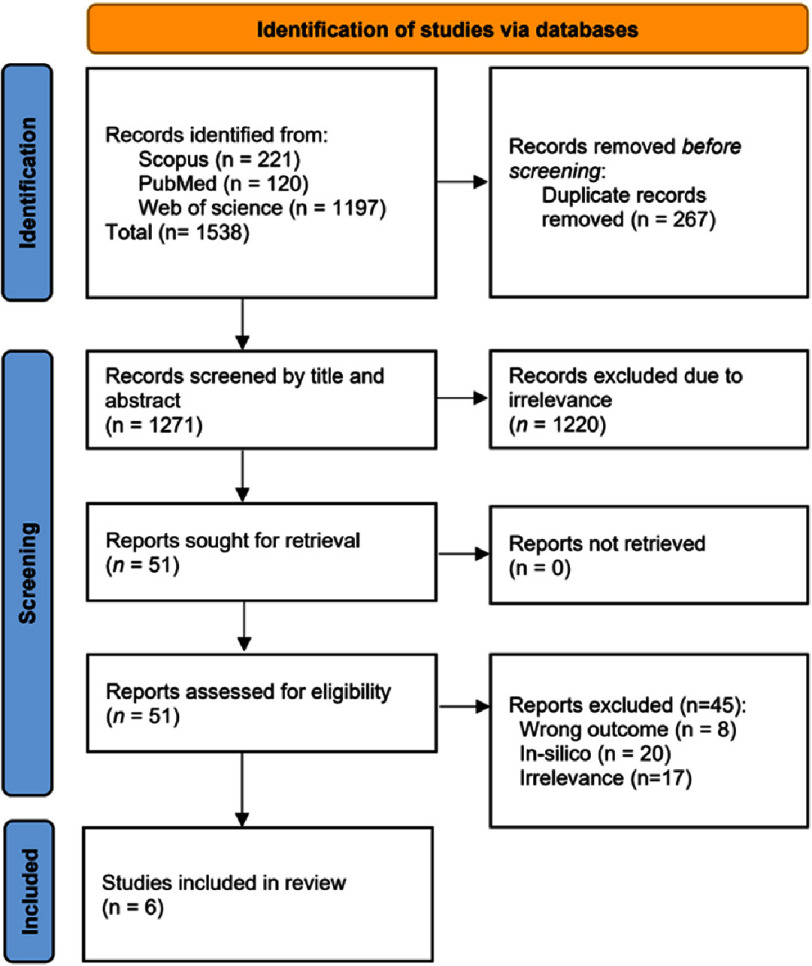
PRISMA flow chart for the selection of studies reporting PCSK9 vaccines in mouse models.

A total of 1,538 records were identified from three databases, namely PubMed (*n* = 120), Scopus (*n* = 221), and Web of Science (*n* = 1,197). After removing 267 duplicate records, 1,271 studies remained for title and abstract screening. Of these, 1,220 reports were excluded for irrelevance based on predefined PECOS criteria, reducing the number of records for full-text retrieval to 51. Among the 51 retrieved reports, 45 were excluded for the following reasons: irrelevant reported outcomes (*n* = 8), only reported results from in silico analysis (*n* = 20), and irrelevant studies (*n* = 9). Finally, six studies were included in this systematic review and meta-analysis for both the qualitative and quantitative analyses. A detailed flowchart of the study selection process is shown in Figure 1.

### Mice model and vaccine design

Data on the vaccine design and the animal model (mice) used to test the vaccine are presented in Table 1. For the mouse model, the ApoE-deficient strain was used in three of the six included studies^25,28,29^. Two studies tested their vaccine efficacy on Ldlr-/- knockout mice, while 1 study tested on C57BL/6-hPCSK9 mice. Nine vaccine designs were reported in the six included studies. A vaccine was designed using human-derived PCSK9-003 epitope peptide with a sequence of F-A-Q-S-I-P-W-N, which was conjugated to Qb virus-like particles (VLPS). Another study used nanoliposomes as the delivery system, where the vaccine itself was prepared from immunogenic-fused PCSK9-tetanus (IFPT)^25,28^. Alhydrogel was added to the vaccine formulation to enhance the immunogenicity of the IFPT, creating an adjuvanted vaccine (IFPTA+)^25,28^. One study used peptides of the C-terminal sequence of PCSK9 with different amino acid segments (580–589 and 682–690 amino acids), which were conjugated to keyhole limpet hemocyanin (KLH) as the delivery system^29^. A vaccine based on PCSK9-mimicking AFFITOPE^®^ was investigated in one study^30^, where it also used KLH as the delivery system^30^. Another study employed the peptide of catalytic domain of human PCSK9 (53–454 amino acid) with D374Y mutation, where the vaccine nanoparticles were prepared with human ferritin protein^26^. The levels of total cholesterol, LDL-C, and triglycerides measured during the follow-up are presented in Table S1.

**Table 1:** Mouse model and vaccine reported in the included studies.

Study	Mice model	Vaccine		
		Name	Peptide and carrier	Delivery system
Wu et al., 2019^31^	Ldlr-/- knockout mice with high-fat diet	PCSK9Qb-003	Human PCSK9-003 epitope peptide (seq.: F-A-Q-S-I-P-W-N)	Qb virus-like particles
Momtazi-Borojeni et al., 2019^28^	ApoE-deficient mice with a T insertion mutation in exon 4.	L-IFPTA+	IFPT peptide with alhydrogel	Nanoliposome
Momtazi-Borojeni et al., 2021^25^	ApoE-deficient mice with a T insertion mutation in exon 4.	L-IFPTA+	IFPT peptide with alhydrogel	Nanoliposome
		IFPT	IFPT peptide	None
		IFPTA+	IFPT peptide with alhydrogel	None
		L-IFPT	IFPT peptide	Nanoliposome
Kawakami et al., 2018^29^	ApoE-deficient mice with a T insertion mutation in exon 4	V1	C-terminal sequence of PCSK9 (580 to 589 aa)	Keyhole limpet hemocyanin
		V2	C-terminal sequence of PCSK9 (682 to 690 aa)	Keyhole limpet hemocyanin
Galabova et al., 2014^30^	Ldlr-/- knockout backcrossed with wild-type C57BL/6J mice	anti-PCSK9	PCSK9-mimicking AFFITOPE^®^	Keyhole limpet hemocyanin
Fang et al., 2024^26^	C57BL/6-hPCSK9 with high-fat diet	PCSK9_NP	Catalytic domain of human PCSK9 (53 to 454 aa, D374Y)	Human ferritin protein

**Notes.**

aa amino acid IFPT immunogenic fused PCSK9-tetanus T thymine

### Quality of the included studies

The overall score of the individual trials on the CAMARADES checklist ranged from 8 to 10 out of 14, indicating moderate-to-high quality (Table 2). However, areas of potential bias were highlighted by the lack of randomization in half of the studies and incomplete reporting of important parameters (such as sample size calculations)^26,29,30^. Furthermore, one study did not explicitly report the blinding protocols used to conceal group allocation^25^. In particular, two studies received low scores^25,31^, suggesting a potentially biased experimental design and reporting transparency (Table 2).

**Table 2:** Results from the appraisal based on CAMARADES criteria.

Criteria	Fang et al., 2024	Galabova et al., 2014	Kawakami et al., 2018	Momtazi -Borojeni et al., 2019	Momtazi -Borojeni et al., 2021	Wu et al., 2019
Publication in peer-reviewed journal	Y	Y	Y	Y	Y	Y
Statement of control of temperature	NM	Y	Y	Y	Y	Y
Randomization to treatment & control	NM	NM	NM	Y	Y	Y
Allocation concealment	Y	Y	Y	Y	NM	Y
Blinded assessment of outcome	Y	Y	Y	Y	Y	Y
Avoidance of Intrinsically neuroprotective anesthetics	NM	Y	NM	Y	NM	Y
Use of animals with cancer	N	N	N	N	N	N
Sample size calculation	Y	NM	NM	NM	Y	NM
Statement of compliance with regulatory requirements	Y	Y	Y	Y	Y	Y
Statement regarding possible conflict of interest	Y	Y	Y	Y	Y	NM
Physiological monitoring	Y	NM	Y	Y	Y	Y
Prespecified Inclusion and exclusion criteria	Y	NM	Y	NM	NM	NM
Reporting animals excluded from analysis	NM	Y	Y	NM	NM	NM
Reporting of study funding	Y	Y	Y	Y	NM	Y
**Total Score (of 14)**	**10**	**10**	**11**	**11**	**9**	**10**

**Notes.**

N no NM not mentioned Y yes

### Effect on total cholesterol

**Figure 2.**
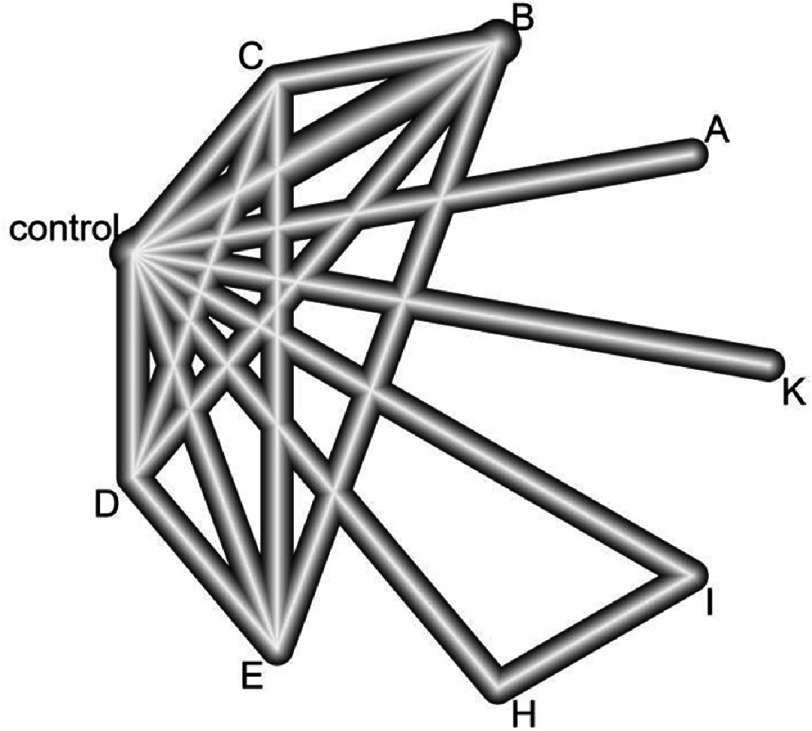
Network graph of the efficacy of PCSK9-targeted vaccines in improving total cholesterol levels. Labels: A, PCSK9Qb-003; B, L-IFPTA+; C, IFPT; D, IFPTA+; E, L-IFPT; F, V1 (dose: 5 µg); G, V2 (dose: 5 µg); H, V1 (dose: 50 µg); I, V2 (dose 50 µg); J, anti-PCSK9; K, PCSK9_NP.

A network graph representing the network meta-analysis of different PCSK9 vaccines, comparing their effectiveness on total cholesterol, is presented in Figure 2. The results of the Bayesian network meta-analysis comparing vaccine efficacy in lowering total cholesterol levels are presented in Table 3. All vaccines had a significant effect on lowering total cholesterol levels (*p*<0.001), except for PCSK9_NP (*p* =0.264). When compared to the control group, V2 (dose: 5 µg) had the best effect (SMD=−7.01 [95%CI:−9.52, −4.50]; *p*<0.001)^29^. The vaccine had similar efficacy to anti-PCSK9 (*p* =0.742) and PCSK9Qb-003 (*p* =0.086)^30,31^.

**Table 3:** Comparison of PCSK9 vaccines based on their efficacy against total cholesterol.

Study	Vaccine	Control as reference		V2_5 µg as reference	
		SMD (95% CI)	*p*-value	SMD (95% CI)	*p*-value
NA	Control	NA	NA	3.92 (0.83 to 7.01)	0.013
Wu et al., 2019^31^	PCSK9Qb-003	−3.09 (−4.90 to −1.28)	<0.001	2.35 (−0.33 to 5.03)	0.086
Momtazi-Borojeni et al., 2019^28^; Momtazi-Borojeni et al., 2021^25^	L-IFPTA+	−4.66 (−5.60 to −3.72)	<0.001	5.4 (2.72 to 8.08)	<0.001
Momtazi-Borojeni et al., 2021^25^	IFPT	−1.61 (−2.54 to −0.67)	<0.001	7.01 (4.50 to 9.52	<0.001
Momtazi-Borojeni et al., 2021^25^	IFPTA+	−2.06 (−3.00 to −1.13)	<0.001	4.95 (2.27 to 7.62)	<0.001
Momtazi-Borojeni et al., 2021^25^	L-IFPT	−3.14 (−4.12 to −2.16)	<0.001	3.87 (1.17 to 6.56)	0.005
Kawakami et al., 2018^29^	V1 (dose: 5 µg)	−5.01 (−7.55 to −2.47)	<0.001	2.00 (0.20 to 3.80)	0.029
Kawakami et al., 2018^29^	V2 (dose: 5 µg)	−7.01 (−9.52 to −4.50)	<0.001	NA	NA
Galabova et al., 2014^30^	anti-PCSK9	−6.50 (−8.25 to −4.74)	<0.001	0.51 (−2.55 to 3.58)	0.742
Fang et al., 2024^26^	PCSK9_NP	−0.83 (−2.29 to 0.63)	0.264	6.1805 (3.2770 to 9.0839)	<0.001

**Notes.**

NA not applicable

**Table 4:** Comparison of PCSK9 vaccines based on their efficacy against low-density lipoprotein cholesterol.

Study	Vaccine	Control as reference		L-IFPTA+ as reference	
		SMD (95% CI)	*p*-value	SMD (95% CI)	*p*-value
NA	Control	NA	NA	2.93 (2.21 to 3.65)	<0.001
Wu et al., 2019^31^	PCSK9Qb-003	−2.35 (−3.82 to −0.88)	0.002	0.58 (−1.06 to 2.22)	0.486
Momtazi-Borojeni et al., 2019^28^; Momtazi-Borojeni et al., 2021^25^	L-IFPTA+	−2.93 (−3.66 to −2.21)	<0.001	NA	NA
Momtazi-Borojeni et al., 2021^25^	IFPT	−0.31 (−1.03 to 0.40)	0.390	2.62 (1.84 to 3.40)	<0.001
Momtazi-Borojeni et al., 2021^25^	IFPTA+	−1.39 (−2.11 to −0.66)	<0.001	1.54 (0.70 to 2.30)	<0.001
Momtazi-Borojeni et al., 2021^25^	L-IFPT	−1.46 (−2.19 to −0.73)	<0.001	1.47 (0.71 to 2.23)	<0.001
Kawakami et al., 2018^29^	V2 (dose: 5 µg)	1.91 (−14.05 to 17.87)	0.815	4.84 (−11.14 to 20.82)	0.553
Kawakami et al., 2018^29^	V2 (dose: 50 µg)	2.86 (14.39 to 20.11)	0.745	5.79 (−11.47 to 23.06)	0.511
Fang et al., 2024^26^	PCSK9_NP	−0.73 (−2.00 to 0.54)	0.261	2.20 (0.74 to 3.67)	0.003

**Notes.**

NA not applicable

### Effect on low-density lipoprotein cholesterol

A comparison of the effectiveness of the developed PCSK9 vaccines is presented in Table 4. Vaccine L-IFPTA+ (*p*<0.001)^25,28^ and PCSK9Qb-003 (*p* =0.002)^31^ were found to have the most significant effectiveness in reducing the LDL-C as compared to control. The SMDs for the two vaccines are −2.35 (95%CI: −3.82 to −0.88) and −2.93 (95%CI: −3.66 to −2.21), respectively. When the vaccine L-IFPTA+ was used as the reference, the effect on LDL-C reduction was not statistically significant in PCSK9Qb-003 (*p* = 0.486). Although a similar effect was also observed for vaccine V2, statistical significance was not observed when the control was used as the reference, even at high concentrations (SMD=2.86 [95%CI: 14.39 to 20.11], *p* =0.745).

### Effect on triglycerides

The effects on serum triglyceride levels of PCSK9 vaccines are compared and presented in Table 5. The reduction in triglycerides was revealed to be significantly effective when vaccines V2 (dose: 5 µg) (*p*<0.001)^29^, L-IFPTA+ (*p* =0.010)^25,28^, and PCSK9Qb-003 (*p* =0.024)^31^ were used. The highest significance was obtained from vaccines V2 (dose: 5 µg) with SMD of −4.90 (−7.36 to −2.45). When the analysis was performed with V2 (dose: 5 µg) as the reference, the effect was found to be similar to of that vaccine PCSK9Qb-003 (*p* =0.158)^31^.

**Table 5:** Comparison of PCSK9 vaccines based on their efficacy against triglycerides.

Study	Vaccine	Control as reference		V2 (dose: 5 µg) as reference	
		SMD (95% CI)	*p*-value	SMD (95% CI)	*p*-value
NA	Control	NA	NA	4.90 (2.45 to 7.36)	<0.001
Wu et al., 2019^31^	PCSK9Qb-003	−2.53 (−4.73 to −0.32)	0.024	2.37 (−0.92 to 5.67)	0.158
Momtazi-Borojeni et al., 2019^28^; Momtazi-Borojeni et al., 2021^25^	L-IFPTA+	−1.56 (−2.75 to −0.38)	0.010	3.34 (0.61 to 6.06)	0.016
Momtazi-Borojeni et al., 2021^25^	IFPT	−0.41 (−1.62 to 0.80)	0.505	4.49 (1.76 to 7.23)	0.001
Momtazi-Borojeni et al., 2021^25^	IFPTA+	−0.97 (−2.18 to 0.24)	0.116	3.93 (1.20 to 6.67)	0.005
Momtazi-Borojeni et al., 2021^25^	L-IFPT	−0.55 (−1.76 to 0.65)	0.368	4.35 (1.61 to 7.09)	0.002
Kawakami et al., 2018^29^	V1 (dose: 5 µg)	0.07 (−1.85 to 2.00)	0.939	4.98 (2.49 to 7.47)	<0.001
Kawakami et al., 2018^29^	V2 (dose: 5 µg)	−4.90 (−7.36 to −2.45)	<0.001	NA	NA
Fang et al., 2025^26^	PCSK9_NP	−0.87 (−2.93 to 1.19)	0.408	4.03 (0.83 to 7.24)	0.014

**Notes.**

NA not applicable

### Network ranking analysis

The ranking scores for each vaccine design compared with the network meta-analysis based on total cholesterol, LDL-C, and triglycerides are presented in Table 6. The first and second top vaccine designs based on total cholesterol were V2 (dose: 5 µg) and Anti-PCSK9, respectively. With a score of 0.959, L-IFPTA+ topped the ranking list for LDL-C outcome, followed by PCSK9Qb-003 with a score of 0.819. In terms of triglyceride clearance, V2 (dose: 5 µg) achieved the top position, followed by PCSK9Qb-003 (score: 0.805). Low confidence in the ranking results was expected in this analysis because of the small number of studies and large standard errors.

**Table 6:** Results from network ranking analysis of the PCSK9 vaccine.

Vaccine	Ranking score		
	Total cholesterol	LDL-C	Triglyceride
PCSK9Qb-003	0.489	0.819	0.805
L-IFPTA+	0.713	0.959	0.702
IFPT	0.227	0.184	0.304
IFPTA+	0.328	0.559	0.517
L-IFPT	0.511	0.593	0.360
V1 (dose: 5 µg)	0.729	NA	0.207
V2 (dose: 5 µg)	0.951	NA	0.988
Anti-PCSK9	0.906	NA	NA
PCSK9_NP	0.131	0.331	0.461
Control	0.0148	0.054	0.156

**Notes.**

NA not applicable

## Heterogeneity and consistency

The results of the heterogeneity and inconsistency tests are presented in Table 7. The data showed relatively low heterogeneity for total cholesterol (*I*^2^ = 16% [95% CI: 0.0% to 57.2%]) and LDL-C (*I*^2^ = 0% [95% CI: 0.0% to 67.6%]). However, the data for triglycerides showed a moderate degree of heterogeneity (*I*^2^ = 64% [95% CI: 26.3%–82.4%]), indicating variability in the effect sizes between the studies. The between-design inconsistency tests did not indicate any significant problems, with most of the *p*-values above 0.05, with the exception of the within-design inconsistency in the LDL-C and triglyceride models, which may indicate the presence of some underlying differences in study design or populations.

**Table 7:** Heterogeneity parameters in each comparison.

Outcome used in the comparison	*I*^2^ (95% CI) (%)	Q-statistics *p*-value		
		Total	Within designs	Between designs
Total cholesterol	16 (0.0 to 57.2)	0.296	0.046	0.712
Low-density lipoprotein	0 (0.0 to 67.6)	0.629	0.045	0.975
Triglycerides	64 (26.3 to 82.4)	0.004	<0.001	0.998

## Discussion

Our results showed that all developed and reported PCSK9 vaccines, except for PCSK9_NP, significantly lowered total cholesterol. As for LDL-C, a significant reduction was found in the groups treated with PCSK9Qb-003, L-IFPTA+, IFPTA+, and L-IFPT. LDL-C was optimally attenuated by L-IFPTA+ and PCSK9Qb-003. Triglyceride reduction was achieved with V2 (dose: 5 µg), L-IFPTA+, and PCSK9Qb-003. The most optimal triglyceride reduction was obtained from the treatment using V2 (dose: 5 µg) and PCSK9Qb-003. Among the reported vaccine designs, PCSK9Qb-003 was consistently superior in improving the total cholesterol, LDL-C, and triglyceride levels. Although V2 (dose: 5 µg) showed slightly larger SMDs for total cholesterol and triglycerides, it did not result in a significant reduction in LDL-C compared to the control, which limits its clinical relevance in the context of PCSK9-targeted therapy. Furthermore, vaccine L-IFPTA+ yielded an optimal reduction in LDL-C, but not in total cholesterol and triglycerides, although the effect was significant when compared to the control. In network ranking analysis, L-IFPTA+ appeared to have the upper hand compared to PCSK9Qb-003, but the results were poor in stability due to the small number of studies and large standard errors of the size effects.

In individual studies, reduced LDL-C and total cholesterol levels were found to be in line with the strong IgG anti-PCSK9 response 9^25,28^. Vaccine PCSK9Qb-003 was reported to downregulate the expression of LDL and very-low-density lipoprotein (VLDL) receptors, which explains that the effect is not only significant in LDL-C reduction, but also in reducing triglycerides^31^. The reduction in serum triglyceride levels might be attributed to the clearance of VLDL because the molecule carries triglyceride as its natural role in transporting lipids from the liver to peripheral tissues. Observing the vaccine effect on triglycerides, especially VLDL, is important because the molecule is converted to LDL following triglyceride hydrolysis and lipolysis in the liver. Elevated LDL is a well-established risk factor for the buildup of cholesterol-rich plaques in the arterial walls^14^. Lower levels of LDL-C reduce the amount of cholesterol available for arterial deposition, which can slow down or even reverse the formation of atherosclerotic plaques^3,14^. This is in line with an individual study reporting vaccine PCSK9Qb-003, in which lipid disposition was attenuated following vaccination^31^.

Vaccines prepared from the PCSK9-003 epitope peptide (F-A-Q-S-I-P-W-N) with Qb virus-like particles (VLPs) have demonstrated superior efficacy in inducing immune responses^29,31^. The PCSK9-003 epitope peptide (F-A-Q-S-I-P-W-N) targets a key region of PCSK9, preventing the degradation of LDL receptors and enhancing LDL-C clearance^32^. Qb VLPs in the PCSK9-003 vaccine act as highly effective delivery systems^31^, enhancing antigen presentation and immune activation by mimicking viral structures^33^. VLP as a vaccine delivery system can be rapidly processed by antigen-presenting cells (APCs) without the need for transcription and translation^34^. In an individual study, the PCSK9Q*β*-003 vaccine led to a 30% decrease in free PCSK9 levels and a notable reduction in aortic lesion area, indicating a decrease in atherosclerotic burden^31^. In addition, VLPs are likely to be safe because they can be derived from non-pathogenic sources such as bacteriophages and plants^35^. The strong immunogenic properties of vaccine V2 are also attributed to its conjugated KLH, which promotes significant T-cell and B-cell activation^29^.

It is worth noting that although both LDL-C and triglyceride outcomes were pooled from studies with non-uniform vaccine dose, follow-up time, and animal models, only triglycerides exhibited moderate heterogeneity (*I^2^* = 64%), whereas LDL-C showed no heterogeneity (*I^2^* = 0%). All included studies treated animals with a high-fat diet, but the mouse strains were different between studies, which could contribute to heterogeneity in the triglyceride response to the vaccines. For example, ApoE-deficient mice exhibit impaired clearance of remnant lipoproteins and VLDL particles, often leading to elevated and variable TG levels even under controlled dietary conditions^25,28,29^. In contrast, LDL-C modulation via the PCSK9–LDLR axis is more directly and uniformly targeted by vaccination, which likely explains the consistent LDL-C-lowering effect observed across studies^5^. Furthermore, differences in baseline insulin sensitivity, hepatic lipogenesis, or lipoprotein lipase activity among these strains may amplify TG variability but not LDL-C. Thus, LDL-C outcomes provide a more reliable and clinically meaningful basis for comparing the relative effectiveness of vaccine formulations.

In contrast, LDL-C outcomes exhibited no observable heterogeneity (*I^2^* = 0%), indicating that the effect of PCSK9 vaccination on LDL-C was highly consistent across the included studies. This low heterogeneity enhances confidence in the pooled estimate and supports the robustness of the vaccine’s effect on this primary lipid parameter. This consistency likely reflects the direct mechanism of action of PCSK9-targeted vaccines, which primarily modulate LDL receptor activity and LDL-C clearance, making LDL-C a more stable and specific endpoint for evaluating vaccine efficacy.

### Limitations of the current study

This is the first study to successfully determine the best PCSK9 vaccine design based on mouse models. Regardless of their promising implications, the findings of this study are limited by several factors. The use of a vaccine in a mouse model limits the generalizability of this study to other species, including humans. However, it is noteworthy that the use of a mouse model is a strategic approach for initial research because of the ease in preparing an atherosclerotic model through genetic modification. The presence of heterogeneity in the analysis suggests that the included studies may differ in terms of study design, animal characteristics, or intervention protocols. Furthermore, the small number of studies and the potential for publication bias are inherent limitations of this meta-analysis.

## Conclusion

In conclusion, vaccine PCSK9Qb-003 demonstrates the most promising efficacy in terms of total cholesterol, LDL-C, and triglyceride reduction. Future studies should explore the vaccine across different species and advance the research to clinical trials once they have been optimized in animal models.

## References

[1] Kobiyama K, Saigusa R, Ley K (2019). Vaccination against atherosclerosis. Current Opinion in Immunology.

[2] Mensah GA, Roth GA, Fuster V (2019). The global burden of cardiovascular diseases and risk factors: 2020 and beyond.

[3] Nettersheim FS, De Vore L, Winkels H (2020). Vaccination in atherosclerosis. Cells.

[4] Verbeek R, Stoekenbroek RM, Hovingh GK (2015). PCSK9 inhibitors: Novel therapeutic agents for the treatment of hypercholesterolemia. European Journal of Pharmacology.

[5] Barale C, Melchionda E, Morotti A, Russo I (2021). PCSK9 biology and its role in atherothrombosis. International Journal of Molecular Sciences.

[6] Zhang X-L, Zhu Q-Q, Zhu L, Chen J-Z, Chen Q-H, Li G-N, Xie J, Kang L-N, Xu B (2015). Safety and efficacy of anti-PCSK9 antibodies: a meta-analysis of 25 randomized, controlled trials. BMC Medicine.

[7] Orringer CE, Blaha MJ, Blankstein R, Budoff MJ, Goldberg RB, Gill EA, Maki KC, Mehta L, Jacobson TA (2021). The National Lipid Association scientific statement on coronary artery calcium scoring to guide preventive strategies for ASCVD risk reduction. Journal of Clinical Lipidology.

[8] Brunham LR, Lonn E, Mehta SR (2024). Dyslipidemia and the current state of cardiovascular disease: epidemiology, risk factors, and effect of lipid lowering. Canadian Journal of Cardiology.

[9] Thanassoulis G, Welsh RC, Hegele RA (2024). What guidelines say about risk reduction: major data on the link between lipid lowering and outcomes. Canadian Journal of Cardiology.

[10] Rached F, Santos RD (2020). The role of statins in current guidelines. Current Atherosclerosis Reports.

[11] Sun L, Wolska A, Amar M, Zubirán R, Remaley AT (2023). Approach to the patient with a suboptimal statin response: causes and algorithm for clinical management. The Journal of Clinical Endocrinology & Metabolism.

[12] Bytyçi I, Penson PE, Mikhailidis DP, Wong ND, Hernandez AV, Sahebkar A, Thompson PD, Mazidi M, Rysz J, Pella D (2022). Prevalence of statin intolerance: a meta-analysis. European Heart Journal.

[13] Zhang B, Chuang G-Y, Biju A, Biner D, Cheng J, Wang Y, Bao S, Chao CW, Lei H, Liu T (2024). Cholesterol reduction by immunization with a PCSK9 mimic. Cell Reports.

[14] Amirfakhryan H (2020). Vaccination against atherosclerosis: An overview. Hellenic Journal of Cardiology.

[15] Matteucci S, Pravatà V, Esposito FM, Pirillo A, Grigore L, Catapano AL (2024). Effect of anti-PCSK9 drugs on the association of PCSK9 to LDL: Anti-PCSK9 Drugs on PCSK9-LDL Association. European Atherosclerosis Journal.

[16] Guo S, Xia X-d, Gu H-m, Zhang D-w (2020). Proprotein convertase subtilisin/kexin-type 9 and lipid metabolism. Lipid Transfer in Lipoprotein Metabolism and Cardiovascular Disease.

[17] Macchi C, Ferri N, Sirtori CR, Corsini A, Banach M, Ruscica M (2021). Proprotein convertase subtilisin/kexin type 9: a view beyond the canonical cholesterol-lowering impact. The American journal of pathology.

[18] Dobó J, Kocsis A, Dani R, Gál P (2022). Proprotein convertases and the complement system. Frontiers in Immunology.

[19] Liu C, Chen J, Chen H, Zhang T, He D, Luo Q, Chi J, Hong Z, Liao Y, Zhang S (2022). PCSK9 inhibition: from current advances to evolving future. Cells.

[20] Kaddoura R, Orabi B, Salam AM (2020). PCSK9 monoclonal antibodies: an overview. Heart Views.

[21] Ugovšek S, Šebeštjen M (2022). Non-lipid effects of PCSK9 monoclonal antibodies on vessel wall. Journal of Clinical Medicine.

[22] Mu G, Xiang Q, Zhou S, Liu Z, Qi L, Jiang J, Gong Y, Xie Q, Wang Z, Zhang H (2020). Efficacy and safety of PCSK9 monoclonal antibodies in patients at high cardiovascular risk: an updated systematic review and meta-analysis of 32 randomized controlled trials. Advances in Therapy.

[23] O’Donoghue ML, Fazio S, Giugliano RP, Stroes ESG, Kanevsky E, Gouni-Berthold I, Im K, Lira Pineda A, Wasserman SM, Češka R (2019). Lipoprotein(a), PCSK9 inhibition, and cardiovascular risk. Circulation.

[24] Toth S, Pella D, Fedacko J (2020). Vaccines targeting PSCK9 for the treatment of hyperlipidemia. Cardiology and Therapy.

[25] Momtazi-Borojeni AA, Jaafari MR, Afshar M, Banach M, Sahebkar A (2021). PCSK9 immunization using nanoliposomes: preventive efficacy against hypercholesterolemia and atherosclerosis. Archives of Medical Science: AMS.

[26] Fang Q, Lu X, Zhu Y, Lv X, Yu F, Ma X, Liu B, Zhang H (2024). Development of a PCSK9-targeted nanoparticle vaccine to effectively decrease the hypercholesterolemia. Cell Reports Medicine.

[27] Zeitlinger M, Bauer M, Reindl-Schwaighofer R, Stoekenbroek RM, Lambert G, Berger-Sieczkowski E, Lagler H, Oesterreicher Z, Wulkersdorfer B, Lührs P (2021). A phase I study assessing the safety, tolerability, immunogenicity, and low-density lipoprotein cholesterol-lowering activity of immunotherapeutics targeting PCSK9. European Journal of Clinical Pharmacology.

[28] Momtazi-Borojeni AA, Jaafari MR, Badiee A, Banach M, Sahebkar A (2019). Therapeutic effect of nanoliposomal PCSK9 vaccine in a mouse model of atherosclerosis. BMC medicine.

[29] Kawakami R, Nozato Y, Nakagami H, Ikeda Y, Shimamura M, Yoshida S, Sun J, Kawano T, Takami Y, Noma T (2018). Development of vaccine for dyslipidemia targeted to a proprotein convertase subtilisin/kexin type 9 (PCSK9) epitope in mice. PLoS One.

[30] Galabova G, Brunner S, Winsauer G, Juno C, Wanko B, Mairhofer A, Lührs P, Schneeberger A, von Bonin A, Mattner F (2014). Peptide-based anti-PCSK9 vaccines-an approach for long-term LDLc management. PLoS One.

[31] Wu D, Zhou Y, Pan Y, Li C, Wang Y, Chen F, Chen X, Yang S, Zhou Z, Liao Y (2020). Vaccine against PCSK9 improved renal fibrosis by regulating fatty acid *β*-oxidation. Journal of the American Heart Association.

[32] Moreno-Gonzalez MA, Ortega-Rivera OA, Steinmetz NF (2023). Two decades of vaccine development against atherosclerosis. Nano Today.

[33] Tariq H, Batool S, Asif S, Ali M, Abbasi BH (2022). Virus-like particles: Revolutionary platforms for developing vaccines against emerging infectious diseases. Frontiers in Microbiology.

[34] Kheirvari M, Liu H, Tumban E (2023). Virus-like Particle Vaccines and Platforms for Vaccine Development. Viruses.

[35] Palma M (2023). Aspects of phage-based vaccines for protein and epitope immunization. Vaccines.

